# Nonfamilial cherubism in a 6-month-old infant: a case report

**DOI:** 10.1186/s12887-024-04825-9

**Published:** 2024-06-20

**Authors:** Seyedeh Sedigheh Hamzavi, Alireza Askari, Rosemina Bahrololoom, Maral Mokhtari, Anahita Sanaei Dashti, Fatemeh Yarmahmoodi, Somaye Rashidi

**Affiliations:** 1 Department of Pediatrics, Nemazee Teaching Hospital, Shiraz University of Medical Sciences, Shiraz, Zand St, Shiraz, Iran; 2 Professor Alborzi Clinical Microbiology Research Center, Shiraz University of Medical Sciences, Shiraz, Iran; 3 Student Research Committee, Shiraz University of Medical Sciences, Shiraz, Iran; 4 Pathology department, Shahid Faghihi Hospital, Shiraz University of Medica Sciences, Shiraz, Iran; 5 Medical Imaging Research Center, Shiraz University of Medical Sciences, Shiraz, Iran

**Keywords:** Bone disease, Cherubism, Familial benign giant-cell tumor of the jaws, Familial multiloculated cystic disease of the jaw, Case report

## Abstract

**Background:**

Cherubism is known as a very rare autosomal dominant familial disorder of childhood caused by a mutation in the SH3BP2 gene on 4p16.3. It has not yet been observed at birth and is usually diagnosed in children aged 2–7. Here, we present a non-hereditary case of cherubism at a very early age.

**Case presentation:**

A 6-month-old girl presented with bilateral progressive jaw enlargement. On physical examination, bilateral asymmetrical jaw enlargement, predominantly on the left side, and some enlarged, non-tender, mobile submandibular lymph nodes were detected. No other abnormality was observed. Further investigations with radiology suggested cherubism and Burkitt’s lymphoma as differential diagnoses. Later on, histopathologic evaluations were suggestive of cherubism. No surgical interventions were indicated, and the child is on regular follow-ups.

**Conclusion:**

Non-hereditary Cherubism, despite scarcity, can present in children below two years of age, even as early as the beginning of primary dentition. Accurate and swift diagnosis is essential to avert physical and psychological complications. Our case report shows the importance of keeping cherubism in mind as a differential diagnosis of bone disease, even in children under a year old, and the value of interdisciplinary collaboration in dealing with rare genetic disorders.

## Introduction

Cherubism is a familial disorder of childhood, first described by William A. Jones in 1933. He named the disorder *Cherubism* due to the cherubic-like appearance, the full round cheeks, and the upward gaze of the eyes in his three patients [1–3]. With only 300 cases reported by 2012 and variable estimated prevalence reported in the literature from 1 per 10,000 to 180,000, overall cherubism is known as a very rare disease with no definite prevalence determined up to date [4–6].

The hallmark of the disease is symmetrical multilocular radiolucent lesions expanding in the mandible and maxilla [4]. This, besides the swelling of the submandibular lymph nodes in the early stages of the disease, contributes to the bizarre shape of the face [7, 8].

Along with multiple visual, dental, respiratory, speech, disfigurement, and psychosocial complications [4], given the disease’s autosomal dominant familial genetic pattern, its incident in the next generation could be a concern for families.

Regarding diagnosis, age, family history, clinical, radiologic, pathology, and molecular evaluations are taken into consideration [4]. Despite that, no firm diagnostic criteria have yet been established, and most treatments used so far have been wait-and-see [9]. Precise diagnosis and management are necessary to prevent inessential treatment and physical and mental complications.

No manifestation of cherubism has been reported at birth; diagnosis is commonly made for children between 2 and 7 years of age, and it is uncommon for patients younger than two years to present with this disorder [4, 10]. Therefore, we aimed to present a non-hereditary case of cherubim with an atypical age presentation with a review of the literature discussing the essential characteristics of approaching this disorder. This case report was conducted according to the CARE guidelines.

## Case presentation

**Fig. 1:**
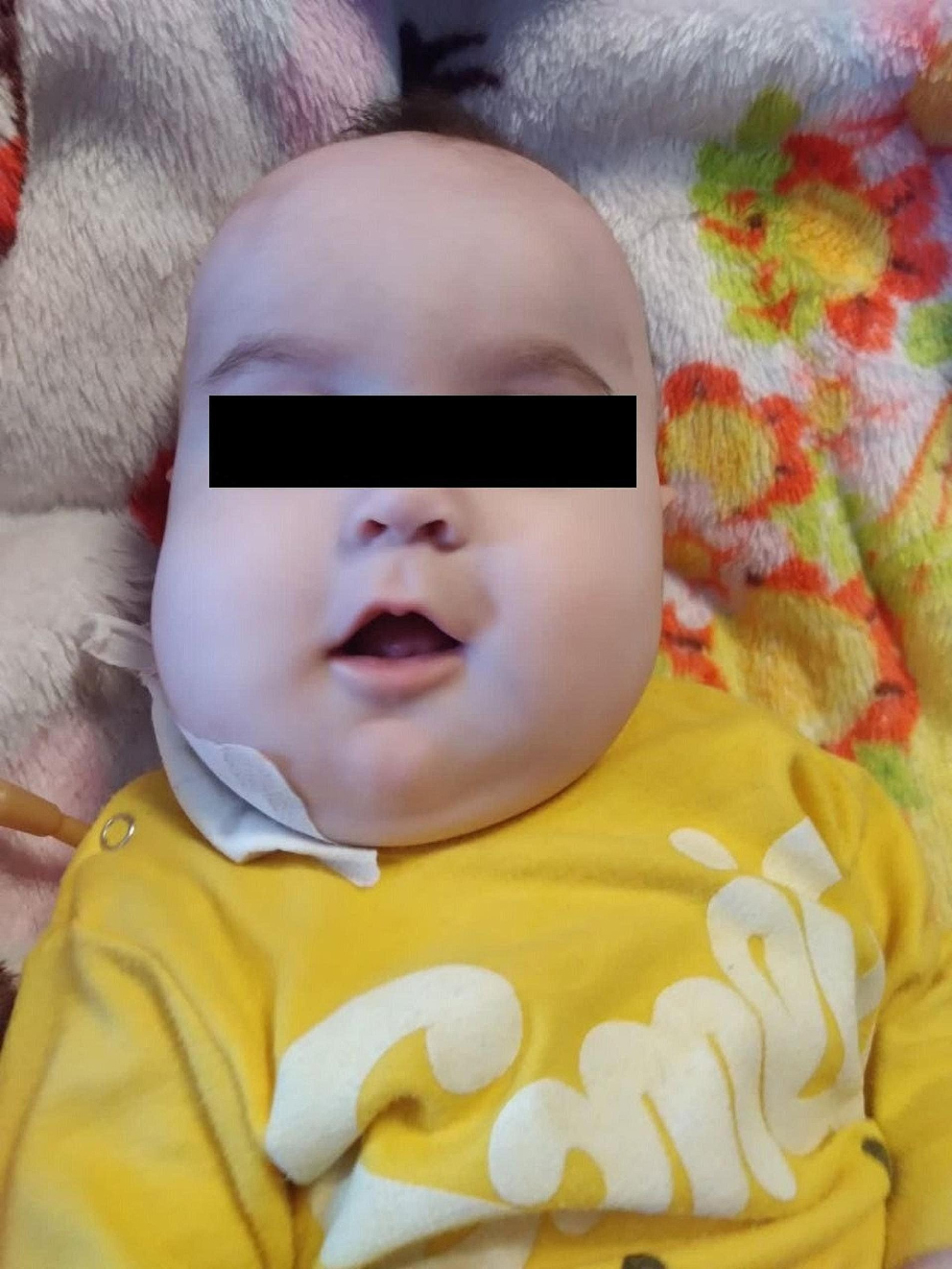
Facial appearance showing an asymmetrical expansion of the mandible

A 6-month-old girl was referred to our university hospital center and was admitted to the pediatric infectious ward with a chief complaint of progressive jaw enlargement (Fig. 1). Her mother had a history of one natural vaginal delivery and two cesarian section pregnancies followed by three live births. She was born via cesarian section and was the third child of the family, with one sibling being unaffected and the other presenting with Sturge-Weber syndrome. There was no history of other familial disorders in the family, and the patient’s development was normal. Her parents noticed the facial disfigurement one month prior to her admission and referred her to a physician, who performed a bone aspiration from the jaw with a suspicion of abscess and administered antibiotics. No fluid or puss was drained, so no pathologic evaluation was possible. She was then referred to our center, Namazi Hospital in Shiraz, Iran, for further evaluation.

On physical examination, bilateral asymmetrical jaw enlargement was detected predominantly on the left side. Her maxilla was not enlarged or deformed, and her eyes were normally placed without upward rolling. Some enlarged, non-tender, mobile submandibular lymph nodes were noticeable, and she had developed a few teeth without any deformities. Cardiovascular, respiratory, neurology, and other examinations were all normal, with no defects in other parts of her body, and she was well-developed and well-nourished.

**Fig. 2:**
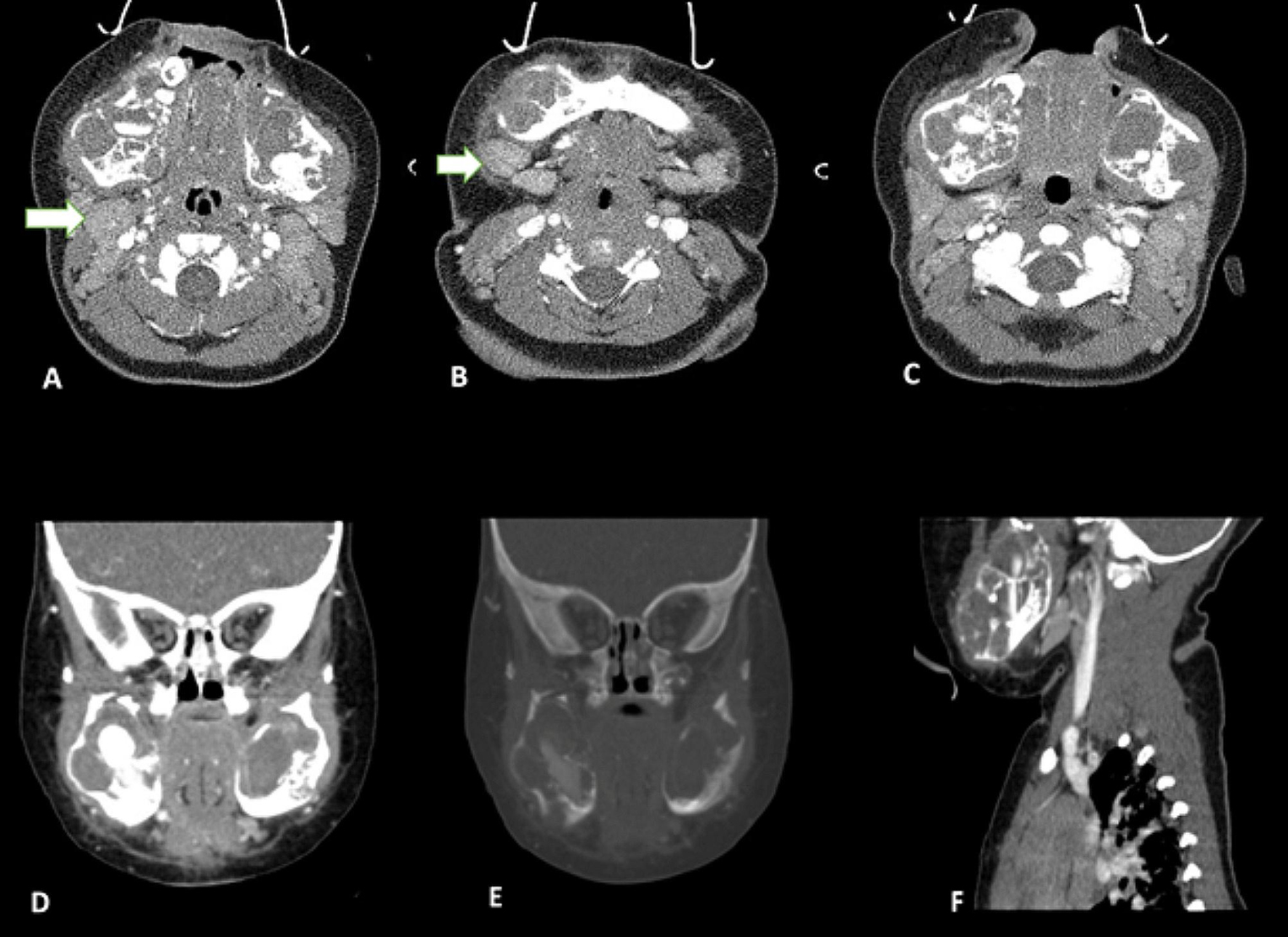
Axial (**A**, **B**, **C**), coronal, bone window (**D**, **E**), and sagittal **F**) images of enhanced CT scan of the face with 3D reconstruction and Intravenous contrast. The images reveal an expansile multi-loculated lytic mass in the body of both mandibular bones without periosteal reaction or soft tissue component, measuring about 37 × 29 × 43 mm on the right side and 34 × 24 × 37 mm on the left side. Both maxillary bones appear normal. Multiple lymph nodes are seen in the submandibular and both jugular chains (white arrow); the largest on the right side measures 15 × 9 mm, and that on the left measures 11 × 7 mm. An enlarged lymph node measuring 15 × 9 mm in the anterior aspect of the trachea

The patient underwent axial, coronal, and sagittal bone window-enhanced computed tomography (CT) with a 3D reconstruction scan of the face, which revealed bilateral expansile multiloculated cystic masses with symmetric involvement of the mandible and sparing of the condyles. Also, multiple enlarged lymph nodes were seen in the submandibular and both jugular chains. Radiologic findings favored Burkitt’s lymphoma and cherubism as differential diagnoses. Figure 2 shows different planes of enhanced CT scans of the head and face. Her chest and abdominal X-rays were reported normal.

**Fig. 3:**
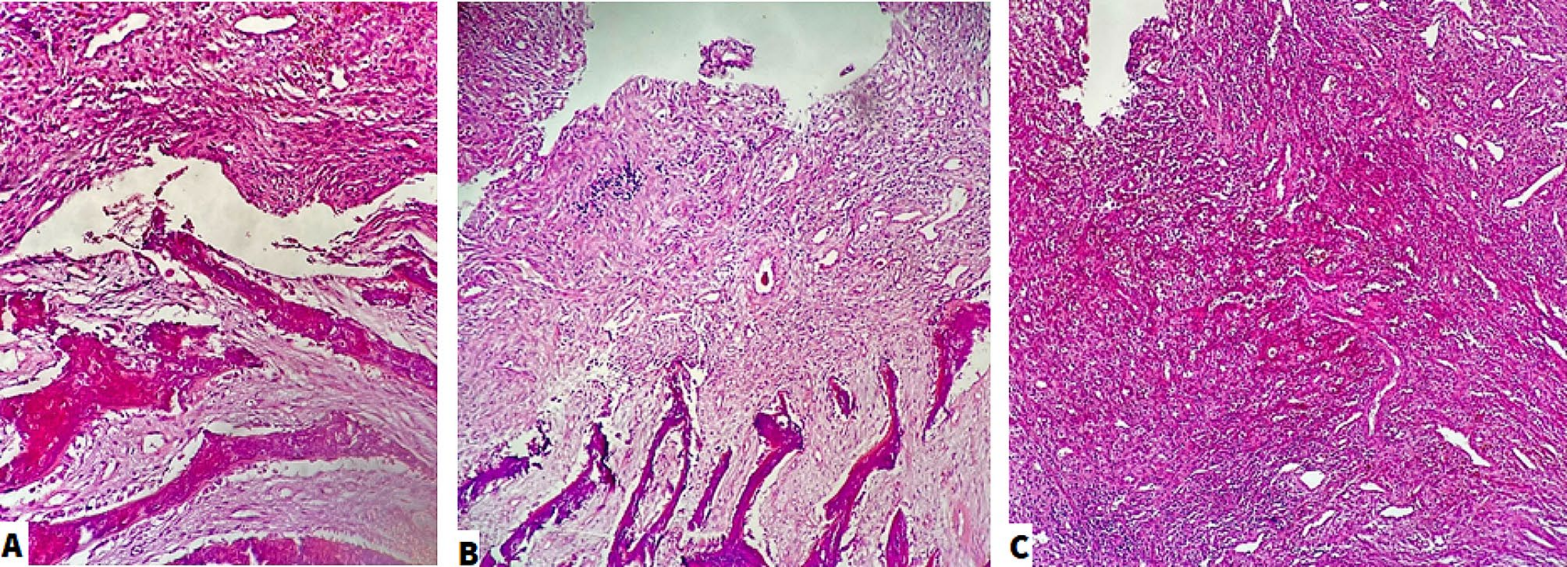
Histopathologic findings of the jaw. **A**: Fibroblast and vascular proliferation, Granulation tissue, and new bone formation, Hematoxylin and eosin stain (H&E), x250. **B**: Fibroblast and vascular proliferation, Granulation tissue, and new bone formation, H&E, x100. C: Inflammation, Red blood cell extravasation, and vascular proliferation, H&E stain, x100

We performed a bone biopsy of the jaw for a definitive diagnosis. As shown in Fig. 3, the biopsy result showed inflammation, Red blood cell (RBC) extravasation, vascular proliferation, Fibroblast, vascular proliferation, Granulation tissue, and new bone formations. Both radiologic and pathologic evaluations were first and foremost in favor of Cherubism. However, formerly other disorders such as Burkitt’s lymphoma, fibrosarcoma, and cyst formations were also thought of as second options. Following this, antibiotic therapy was terminated.

The patient’s laboratory test results showed an average white blood cell count of 8300/µL and an increased platelet count of 866,000 on admission, followed by 425,000 and 520,000 on discharge. The peripheral blood smear test was reported as normal. The erythrocyte sedimentation rate (ESR) was 8 mm/hr, while the C-reactive protein (CRP) level was 10 mg/L. Furthermore, the patient presented with a blood urea nitrogen (BUN) level of 8 mg/dL and a creatinine (Cr) level of 0.36 mg/dL, which were all normal. Other laboratory data indicated an increased lactate dehydrogenase (LDH) level of 757 IU/L, an average uric acid level of 3.4 mg/dL, and an average ferritin level of 364 ng/mL. The prothrombin time (PT) was measured at 16 s, the partial thromboplastin time (PTT) at 32 s, and the international normalized ratio (INR) at 1.26, all in the normal range. No other laboratory evaluations and no genetic testing were done.

With clinical, radiological, and histological findings, grade I cherubism was diagnosed soon. No new medications were administered after the diagnosis, and antibiotic administration was terminated. She was then referred to a maxillofacial surgeon who planned a regular follow-up due to her mild involvement. She is now 14 months old, and no progression has been reported in her follow-ups since.

## Discussion

Cherubism (OMIM #118,400) is classified by the World Health Organization (WHO) as a non-neoplastic bone lesion that exclusively affects the jaws [11] followed by an autosomal dominant mutation pattern with variable expressions of a gene on chromosome 4 that encodes SH3-domain binding protein 2, also known as SH3BP2 [4]. 20% of patients might not have this recognizable gene mutation pattern [12] and according to Rezende et al., male children are more prone to this mutation [13].

In affected children, skeletal dysplasia and fibro-osseous lesions usually appear in their first or second decade of life. In most cases, the progression of dysplasia tends to recede when the patient reaches puberty. Although lesions are usually asymptomatic and limited to the mandibular and maxillary bones, in severe cases, a misdiagnosis or late treatment may lead to the invasion of lesions to the orbital fossa, infrequently upper respiratory tract obstruction, and disruption of primary dentition along with inflammation and facial deformity leading to psychosocial and quality of life implications especially for young children [4, 7, 8].

Based on the regional extension of the lesion, cherubism was first suggested to be classified into three grades by Fordyce in 1976 [14] which was later upgraded to six grades. Grade I, only involvement of the mandible, grade II, involvement of both the mandible and the maxilla, grade III, aggressive involvement of the mandible with root resorption, grade IV, aggressive involvement of both the mandible and the maxilla with root resorption, grade V, progressive, aggressive and extending lesions involving both the mandible and the maxilla and occasionally the coronoid and the condyles causing facial disfigurement, grade VI, progressive, aggressive and spreading lesions involving the orbital fossa additional to all the areas mentioned before [4]. According to this classification, our case falls under grade I.

Benign bone conditions like giant cell granuloma, fibrous dysplasia, juvenile ossifying fibroma, fibrous osteoma, hyperparathyroidism, and malignant bone lesions like osteosarcoma can be considered important differential diagnoses for cherubism [4, 15]. Regarding the history of clinically diagnosed Sturge-Weber syndrome (SWS) in the family, since the association of SWS with maxillofacial and mandibular osseous abnormalities has been previously reported [16] this question was brought up whether there could be an association between the genetic patterns of cherubism and SWS; which no descriptions were made in the literature regarding this subject.

Computed tomography (CT) and magnetic resonance imaging (MRI) are frequently used to diagnose and manage cherubism; CT imaging is a more sensitive modality [17, 18].

Pathology also plays an essential role in the diagnosis of this disorder. Cherubism can mimic other bone pathologies; therefore, genetic testing is known as the best substantiate tool for diagnosis. However, some experts have considered tissue biopsy accompanied by clinical and radiological findings sufficient for diagnosis confirmation [19]. No genetic testing was done for our case due to her short duration of admission and the family’s inability to meet the costs. Hence, diagnosis was based on clinical, radiologic, and pathological evaluations, which could be the limitation of our study.

Regarding laboratory test findings, while most biochemical markers have been reported to be typically within a normal range, alkaline phosphatase, TNF-α urine calcium/creatinine, and other bone remodeling markers might be elevated [4, 13, 20]. Similarly, in our case, except for an elevated LDH and PLT, other laboratory findings were in the normal range. However, Calcium, phosphorus, alkaline phosphatase, and TNF-α were not assessed in our patients, which could be another limitation of this study.

While generally, no treatment is necessary as the mass tends to disappear with the child reaching puberty, in severe cases with functional disability, invasive treatment like surgery or radiotherapy has been suggested. Likewise, our patient had mild involvement and is under longitudinal observation [21, 22]. Surgery is not recommended in mild cases due to the possibility of recurrence. Although Steroids, TNF-α inhibitors, calcitonin, anti-neoplastic agents, immunosuppressants, and monoclonal antibodies have been reported as an option for the pharmacological treatment of cherubism, none have yet been confirmed by clinical trials as a possibility for effective treatment [22–24].

## Conclusion

To our knowledge, this is the first reported case of cherubism in a 6-month-old infant.

Non-hereditary Cherubism, despite scarcity, can present in children below two years of age, even as early as the beginning of primary dentition.

Although treatment options for cherubism remain limited, understanding this condition’s clinical and histological features can help clinicians provide appropriate care and counseling to affected patients and their families, thus preventing false diagnoses and unnecessary investigations, especially in young patients like ours. Further research and long-term follow-up are needed to understand cherubism’s pathogenesis better and develop new therapeutic approaches. Overall, our case report indicates the importance of having cherubism in mind as a differential diagnosis of bone disease, even in children under a year old, and highlights the value of interdisciplinary collaboration in managing rare genetic disorders.

## Data Availability

The datasets regarding our participant’s laboratory, radiologic, and clinical findings during the current study are available from S.hamzavi55@yahoo.com upon reasonable request.

## Abbreviations

CXR: Chest X-ray
CT: Computed Tomography
SWS: Sturge-Weber syndrome
MRI: Magnetic resonance imaging
TNF-α: Tumor necrosis factor-α
LDH: Lactate dehydrogenase
PLT: Platelet
WBC: White blood cell
RBC: Red blood cell
ESR: Erythrocyte sedimentation rate
CRP: C-reactive protein
PT: Prothrombin time
PTT: Partial thromboplastin time
BUN: Blood urea nitrogen
Cr: Creatinine
INR: International normalized ratio
